# Traumatic Brain Injury and the Road to Alzheimer’s Disease

**DOI:** 10.3390/biomedicines14092056

**Published:** 2026-09-13

**Authors:** Roxana Kaveh, Farzin Kamari, Poul Flemming Høilund-Carlsen, Morten Blaabjerg, Alex Alban Christensen, Frantz Rom Poulsen, Sarvenaz Ghaedi, Abass Alavi, Sasan Andalib

**Affiliations:** 1 Guilan Road Trauma Research Center, Trauma Institute, Guilan University of Medical Sciences, Rasht 4193713194, Iran; rxnkaveh@gmail.com; 2 Institute of Physiology, Department of Neurophysiology, Eberhard Karls University of Tübingen, 72074 Tübingen, Germany; farzin.kamari@uni-tuebingen.de; 3 Department of Clinical Research, Faculty of Health Sciences, University of Southern Denmark, 5230 Odense, Denmark; pfhc@rsyd.dk; 4 Department of Nuclear Medicine, Odense University Hospital, 5000 Odense, Denmark; 5 Research Unit of Neurology, Department of Clinical Research, Faculty of Health Sciences, University of Southern Denmark, 5230 Odense, Denmark; morten.blaabjerg1@rsyd.dk (M.B.); alex.alban.christensen@rsyd.dk (A.A.C.); 6 Department of Neurology, Odense University Hospital, 5000 Odense, Denmark; 7 Department of Neurosurgery, Odense University Hospital, 5000 Odense, Denmark; frantz.r.poulsen@rsyd.dk; 8 Brain Research Interdisciplinary Guided Excellence (BRIDGE), University of Southern Denmark, 5230 Odense, Denmark; 9 Independent Researcher, Lake Forest, CA 92610, USA; sarvenazghaedi@gmail.com; 10 Department of Radiology, Perelman School of Medicine, University of Pennsylvania, Philadelphia, PA 19104, USA; abass.alavi@pennmedicine.upenn.edu

**Keywords:** traumatic brain injury, Alzheimer’s disease, neuroinflammation, amyloidopathy, tauopathy

## Abstract

Alzheimer’s disease (AD) is associated with both mild and moderate to severe traumatic brain injury (TBI). This narrative review gives an account of the association of TBI and AD and highlights possible cellular and molecular pathways linking these pathologies. Following TBI, the immune system of the brain is rapidly activated and gives rise to acute neuroinflammation. While neuroinflammation is protective in nature, it may persist chronically in the case of less-controlled prolonged responses, triggering neuroprotective loss and neurotoxicity. Moreover, reduced clearance of amyloid-beta may occur, along with its overproduction and aggregation. Tau protein regulation is also altered by kinase and phosphatase enzymes, resulting in the accumulation of hyperphosphorylated tau protein in neurons and glial cells and the emergence of intracellular tau neurofibrillary tangles. More to the point, vascular impairment following TBI has been reported to contribute to cognitive decline and AD. Blood–brain barrier breakdown following TBI allows for infiltration of peripheral immune cells and blood-derived proteins into the brain, which exacerbates neuroinflammation, interrupts synaptic signaling, and promotes oxidative stress. The neuroinflammatory response, dynamic alterations in amyloid and tau biology, and vascular impairment are thought to interact within a broader network of processes associated with AD neurodegeneration, rather than acting as isolated mechanisms.

## 1. Introduction

Traumatic brain injury (TBI) is a major public health burden, affecting approximately 38 million existing cases globally [1]. The complex TBI pathophysiology causes substantial clinical and societal challenges [2]. Notably, TBI is suggested as a significant risk factor for the subsequent development of neurological and psychiatric disorders, including neurodegenerative disorders such as Alzheimer’s disease (AD) [3]. While the acute TBI outcomes are well addressed throughout the literature, there is less information about potential long-term neurodegenerative sequelae.

The possible chronic neurodegenerative outcomes of TBI were first described in professional National Football League players exposed to repetitive mild TBI [4]. Later, evidence suggested an association between a history of TBI and an increased risk of later-life dementia [5,6] and possibly AD [7]. AD is a neurodegenerative disorder and the most prevalent type of dementia. Although the exact causes remain unknown, AD is characterized by pathophysiological alterations, including the accumulation of Amyloid-β (Aβ), hyperphosphorylated neurofibrillary tangles of tau protein (NFTs), and widespread neural loss.

There is evidence to suggest that a complex interplay of mechanisms, including inflammatory responses, Aβ accumulation, modifications of tau biology, and vascular disruption, is involved in the pathophysiological responses secondary to TBI. Prolonged activation of these pathological processes appears to contribute to neurodegeneration. Thus, TBI should not be viewed as a fixed, one-time injury to the brain, but rather as a dynamic, potentially progressive condition with long-term pathological consequences. This relationship appears to be complex and multifactorial, depending on the frequency and severity of the injury, anatomical location, age, sex, and the individual’s genetic susceptibility [8]. Of note, TBI severity can be classified through several grading systems, ranging from mild to moderate to severe. Most of the grading systems are based on acute injury specifications and acute symptoms, such as baseline consciousness status and progression, and do not account for chronic sequelae.

It should be noted that AD is not the only tau-related disease that can develop following TBI; chronic traumatic encephalopathy (CTE) or other proteinopathies in the brain might occur as well. In a case series of athletes exposed to repetitive head injuries who died before the age of 30, CTE neuropathology was shown in approximately 40% of cases [9]. In this study, CTE was evidenced by perivascular phosphorylated tau aggregates, often, as well as other pathologies, including ventricular enlargement, cavum septum pellucidum, thalamic notching, and perivascular pigment-laden macrophage aggregation in the frontal white matter. Another study reported CTE neuropathology in a high proportion of former football players, 85% of whom with severe CTE showed signs of dementia [10]. Furthermore, in retired football (soccer) players who developed dementia later in life, mixed pathologies were detected, including those of CTE, AD, and TAR DNA-binding protein 43 (TDP-43) [11]. Nevertheless, an important gap remains in the literature in distinguishing between TBI-related CTE and AD. While a CTE diagnosis requires post-mortem histopathological findings [12], a set of diagnostic criteria for the associated clinical disorder, traumatic encephalopathy syndrome, has been proposed to identify post-TBI living patients who are likely to suffer from CTE [12]. CTE is neuropathologically defined by consensus criteria as hyperphosphorylated tau accumulation within neurons and astrocytes, distributed irregularly near the small blood vessels at cortical sulcal depths [13]. In addition, CTE has been associated with cortical neurodegeneration, particularly in the sulci [14]. This preferential sulcal vulnerability, especially at depth, is also seen in AD. Higher tau accumulation in sulci than in gyri, together with higher lifelong hippocampal connectivity in these regions, are suggested as possible contributors to the sulci vulnerability pattern in AD [15]. Tau pathology in AD is reported to progress according to an order of six stages, beginning in the transentorhinal region, progressing to the entorhinal cortex and hippocampus, and finally affecting the isocortex [16]. In CTE and in CTE with AD neuropathological change, tau pathology was found consistently in the upper cortical layers, similar to the pattern observed in tauopathies [17]. In addition, in CTE neuropathology, astroglial tau appeared to be distributed preferentially in the cortical sulci rather than the gyri, whereas the neuronal tau pathology was distributed more evenly from the gyral crest to the sulcal depth, a pattern comparable to that seen in AD [18]. In a study using immunofluorescence analysis, both CTE and AD demonstrated mixed 3-repeat and 4-repeat tau isoforms in the temporal cortex, with 4R tau dominating in mild disease and shifting toward 3R as severity increased [19]. However, with respect to hippocampal subfield accumulation, CTE showed a greater burden of tau in CA4 and CA2/3, whereas AD showed greater relative involvement of CA1 and the subiculum. Tau immunophenotyping indicated that the astroglial and neurofibrillary tau pathologies of CTE are distinct [20]. Astrocytic tau pathology resembled that of aging-related tau astrogliopathy, containing 4-repeat tau, while neurofibrillary tangles of CTE mirrored those of AD and primary age-related tauopathy, containing both 3-repeat and 4-repeat tau with comparable post-translational modifications and conformations [20]. Thus, the distinction between AD and CTE appears to rely more on the pattern and distribution of tau pathology than on its immunophenotype. Biomarkers may also be useful in distinguishing these two conditions. Analysis of post-mortem cerebrospinal fluid (CSF) samples from patients with autopsy-confirmed diagnoses demonstrated increased phosphorylated tau231 and decreased Aβ1-42 levels in the CTE group compared with the AD group and with those with neither pathology [21]. Another study found increased plasma phosphorylated tau181 and tau217 in traumatic encephalopathy syndrome patients with AD pathology, and the lowest phosphorylated tau levels in autopsy-confirmed CTE cases without AD pathology [22].

This narrative review gives an account of the association between TBI and AD and the underlying cellular and molecular mechanisms.

## 2. Methods

The literature search for this narrative review was performed on English-language studies up to August 2026, using PubMed and Google Scholar. In the literature search, terms such as traumatic brain injury, head injury, Alzheimer’s disease, dementia, neurodegeneration, amyloid-beta (Aβ), tau, neurofibrillary tangles, neuroinflammation, blood–brain barrier disruption, and vascular injury were used, individually or in combination. Publications were thereafter selected according to their relevance and contribution to the overall narrative of the review.

## 3. Evidence for the Possible Link Between TBI, Dementia, and AD

### 3.1. The Association of TBI with the Later Risk of AD and Dementia

Several studies provide evidence regarding the association between TBI and later development of neurodegenerative disorders, including AD. A meta-analysis concluded a possible association between a history of TBI and an increased risk of developing AD, particularly when moderate to severe [23]. Moreover, it has been demonstrated that a history of TBI increases the risk of all-cause dementia in individuals aged over 50 [5] and in veterans [6]. Former male amateur athletes who participated in soccer, boxing, and wrestling were shown to have an increased risk of dementia compared with the general population in a systematic review and meta-analysis [24]. The risk appeared to be stronger among retired professional soccer and American football players than among amateur players. An increased risk of dementia has also been demonstrated in association with head injury in a prospective community-based cohort study with 25 years of follow-up, with the association appearing to be dose-dependent, increasing with injury frequency [25]. Moreover, the association of TBI with dementia was found to be more pronounced when TBI occurred between ages 50 and 69, and this association did not appear to be explained by genetic or early life environmental factors [26]. Mild TBI has also been reported to be likely associated with an elevated risk of dementia [27] and AD [7]. In a study in older-aged veterans (mean age of roughly 68 years), TBI history was found to be linked to a higher 9-year risk of dementia by approximately 60%, with dementia onset occurring almost 2 years earlier in veterans with TBI compared to those without TBI [28]. Data from an investigation in an emergency department and inpatient visits showed an increased risk of dementia development in mild TBI in cases aged 65 or more, or moderate to severe TBI in cases aged 55 or more [29]. Moreover, younger adults were observed to be potentially less vulnerable to the post-TBI effects following a recent mild trauma. Thus, the post-TBI increased risk of dementia appears to be related to both the age of the individuals at the time of injury and the injury severity. Assessment of the time- and dose-dependent risk of dementia following TBI revealed an elevated risk of dementia even decades after TBI, especially in individuals with multiple or more severe TBI, although the risk weakened over time [30]. Another study found that TBI occurring after mild cognitive impairment diagnosis modestly increased the subsequent risk of AD progression, as well as that of behavioral and psychological symptoms of dementia onset after a diagnosis of AD dementia [31]. However, TBI severity only affected the risk of AD progression and not the risk of developing behavioral and psychological symptoms of dementia. Moreover, individuals under the age of 65 demonstrated a higher risk of progression to AD dementia. The literature supporting an increased risk of all-cause dementia after TBI is more balanced than that supporting the link between TBI and AD. An umbrella meta-analysis supported TBI as a significant risk factor for dementia, demonstrating that dementia risk increases with TBI severity, but concluded that its association with AD remains unclear [32]. A population-based study found stronger associations of TBI with the risk of vascular dementia and unspecified dementia than with that of AD [33]. A systematic review and meta-analysis also demonstrated an association between TBI and an elevated risk of dementia, but reported no association with AD specifically [34].

### 3.2. Reverse Causality and Confounding Factors

An important consideration when interpreting the relationship between TBI and AD is reverse causality, meaning that cognitive decline may itself predispose individuals to fall and thereby increase their susceptibility to head injury. Cognitive and physical frailty was shown to be a significant predictor of falls among older adults [35]. With respect to reverse causality, a cohort study demonstrated a four- to six-fold increased risk of a dementia diagnosis within the first year after TBI, after which the risk fell rapidly but remained present for more than 30 years [30]. To explain the first-year greater association, the study discussed that some of the individuals might have had prior executive function impairment, which made them more likely to experience TBI. In agreement with this evidence, a retrospective cohort study of individuals aged over 65 with traumatic injury and no prior diagnosis of dementia showed that a history of falls was followed by a diagnosis of AD or a related dementia in 10.6% of patients within one year [36]. Moreover, in older adults, falls significantly increased the risk of dementia [37]. It appears that the relationship between TBI and dementia or AD is not simply unidirectional, and reverse causality should be considered. Another complication regarding this association is that evidence has also shown increased risk of falls after head injury in community-dwelling older adults over a follow-up of up to 30 years, which appeared to be stronger for more frequent and more severe injuries [38]. These findings point to a cycle of events which should be considered when interpreting the association between TBI and dementia or AD.

Evidence also demonstrates several other factors that may contribute to the relationship between TBI and dementia or AD. A cohort of almost 3.8 million children and adolescent inpatients demonstrated that alcohol use disorder raised the risk of TBI-related hospitalization [39]. Dementia risk was also shown to be increased in heavy alcohol drinkers, whereas it was reduced in those who maintained mild-to-moderate alcohol consumption [40]. Another study reported an increased risk of dementia associated with alcohol use but found no evidence of a protective effect of moderate alcohol consumption [41]. Because alcohol use is possibly associated with both TBI and dementia, studies examining the association of TBI with dementia or AD should consider adjusting for alcohol use. Supporting this finding, a study reported an increased incidence of dementia in individuals hospitalized for major TBI, although the risk was attenuated after adjustment for confounders, particularly alcohol consumption and physical activity [42]. Another factor that should be considered in the association between TBI and dementia or AD is frailty, which was shown to be prevalent in older individuals with TBI [43] as well as a significant independent risk factor for all-cause dementia [44]. A population-based cohort study found an association between a higher cardiovascular risk burden (Framingham General Cardiovascular Risk Score) and an elevated risk of dementia and Alzheimer’s dementia [45]. A meta-analysis also reported an association between hypertension and an increased likelihood of AD [46]. With respect to TBI, orthostatic hypotension, specifically changes in diastolic blood pressure, was identified as an independent risk factor for falls among community-based middle-aged adults over two decades of follow-up [47]. These findings suggest that vascular disease may contribute to both conditions and should therefore be considered as a potential confounding factor for the TBI and dementia or AD association. Depression perhaps provides the clearest example of the complex relationship between reverse causality and confounding factors in the literature regarding TBI and AD association. A population-based prospective cohort study of adults aged 45 and over showed a positive association between depressive symptoms and an increased risk of falls during three years of follow-up [48]. A diagnosis of depression was also associated with more than twice the risk of dementia in both sexes [49]. On the other hand, a systematic review and meta-analysis found that the history of TBI nearly doubled the risk of developing depressive symptoms, compared with individuals without such a history [50]. Another systematic review and meta-analysis likewise pointed to the high prevalence of depression among individuals with mild cognitive impairment [51]. Depression is only one example, and other psychiatric conditions may show comparable associations.

## 4. Cellular and Molecular Mechanisms Linking TBI and AD

### 4.1. Neuroinflammation

The microglia-mediated inflammatory response following TBI is thought to act as a double-edged sword, contributing to both tissue repair and secondary injury, depending on its regulatory balance [52]. Hence, it appears that neuroinflammation may be crucial for determining TBI outcomes.

#### 4.1.1. Microglia Activation and Polarization

TBI activates innate immunity and inflammation through the release of damage-associated molecular patterns from stressed cells, which are subsequently recognized by Pattern Recognition Receptors, including Toll-like receptors and Nucleotide-binding Oligomerization Domain-like receptors. These receptors are broadly expressed on various brain cell types, such as microglia, astrocytes, and neurons, and are upregulated following the injury [53]. Peripheral immune cells, including neutrophils, are also observed to infiltrate the injury site post-injury [54].

It was demonstrated that early inflammation, which happens within the first 24 h post-injury, is largely independent of microglia, while later phases (7–30 days post-injury) are proposed to be microglia-dependent, highlighting the crucial role of microglia in the chronic pathology of TBI [55]. A study observed activation of microglia within minutes of diffuse TBI [56]. However, the activated microglia were restricted to the damaged (membrane-permeabilized) neurons and surrounding areas only. Beyond microglia, single-cell analysis of the mouse hippocampus revealed other cells associated with TBI [57]. The study demonstrated marked astrocyte activation and population expansion after TBI. The astrocyte inflammatory response-related pathways were enhanced in TBI samples, along with microglia transcriptional changes, indicative of their co-contribution in acute phase TBI inflammatory responses.

Following ligand recognition, microglia are activated and are further polarized into at least two phenotypes, M1-like (pro-inflammatory) and M2-like (anti-inflammatory). Single-cell RNA sequencing revealed that after TBI, proliferating microglia dominantly exhibit an M1-like phenotype, which is suggested to have a central role in regulating the complex cellular network of neuroinflammatory responses following TBI [58]. M1-like microglia release cytokines (e.g., Interleukin-1 (IL-1), IL-6, IL-12), chemokines, and tumor necrosis factor alpha that contribute to neuronal damage. M1 also expresses Nicotinamide Adenine Dinucleotide Phosphate (NADPH) and induces nitrite oxidase synthesis, further enhancing the neurotoxicity. M2-like microglia, on the other hand, release IL-4, IL-10, and IL-13, as well as transforming growth factor-beta, thereby promoting tissue repair and limiting inflammation. These microglia/macrophage responses are dynamic, with early increases in both M1 and M2 phenotype markers. The M2 phenotype increase appears to be transient and thus subsequently declines, while the M1 response possibly persists and correlates with the extent of white matter degeneration [59]. A review article mentioned that the post-TBI released cytokines show temporal functional pleiotropy, meaning that the same cytokines may exhibit different, even opposed, functions depending on when they act following a TBI, in either acute, sub-acute, or chronic phases [60]. The timeframe of different activated microglia states and the temporal pattern of the released cytokines seem important determinants of the therapeutic effects of anti-inflammatory interventions in experimental studies and should be considered when translating these findings into clinical practice. An experimental study explored post-TBI inflammatory responses, including microglia and monocyte infiltration, in the presence of early Aβ accumulation in AD-transgenic mouse models, at 3 and 120 days following TBI [61]. The results at 3 days post-TBI demonstrated attenuated inflammatory responses in AD transgenic mice compared to non-transgenic mice. At 120 days post-injury, the neuroinflammatory responses following TBI were altered by shifting to a more persistent chronic response in the transgenic mice. Aβ plaque formation or tau pathology was not significantly affected by TBI. Another study reported changes in post-TBI neuroinflammatory responses in age-related AD mouse models [62]. Compared to wild-type mice, AD models demonstrated a delayed but persistent inflammatory response following injury. Delayed glial activation was consistently documented in transgenic AD mice subjected to TBI, in association with worsened Aβ accumulation compared to uninjured AD mice [63].

It should be noted that the traditional binary M1/M2 concept of microglial polarization is oversimplified and does not fully capture microglial heterogeneity. Single-cell analysis of microglia across the mouse lifespan and in the injured brain demonstrated microglial heterogeneity, particularly at young ages, and several distinct activated microglial subtypes in demyelinating lesions [64]. Following ischemic injury in mice, microglia and macrophages were shown to polarize into different phenotypes with distinct patterns, depending on signals from the surrounding microenvironment [65]. Significant microglial remodeling and several microglial subgroups were also identified in mouse models of moderate controlled cortical impact, shifting preferentially from a regenerative phenotype toward pro-inflammatory and disease-associated states [58]. Single-cell analysis of TBI and normal brains from both wild-type mice and mice genetically lacking macrophage influx (CCR2^−/−^) revealed distinct microglial subsets in acute TBI and in normal brains [66]. The study demonstrated that the absence of CCR2 modified the transcriptional profiles of microglial subsets and suggested an influence of infiltrating monocytes and macrophages on microglial activation. This finding is important, since monocyte-derived macrophages are also involved in neuroinflammatory responses following TBI. These findings highlight the complexity of microglial polarization, which should be considered when interpreting the data.

Multiple signaling pathways regulate the heterogeneous microglial phenotypic transition after TBI. A study in a rat controlled cortical impact model proposed that the ephrin receptor A4/mitogen-activated protein kinase axis possibly activates endoplasmic reticulum stress-related proteins, promoting M1 polarization of microglia and the progression of TBI [67]. Pharmacological inhibition of ephrin receptor A4 with the KYL peptide, on the other hand, attenuated M1 polarization and the progression of TBI. Another study described myeloid-related immune responses following TBI, showing dynamic tissue-specific cellular composition, transcriptional programs, and activation states [68]. Furthermore, the study identified three major ligand–receptor signaling axes of Ccl2/Ccl7–Ccr2, Tnf–Tnfrsf1b, and Grn–Flna potentially mediating monocyte recruitment, M1 polarization, and macrophage differentiation.

Peroxisome proliferator-activated receptor gamma activation, on the other hand, is observed to promote microglia towards an M2-like phenotype, increasing anti-inflammatory activity, as well as minimizing axonal damage [69]. Additionally, signal transducer and activator of transcription (STAT) pathways affect polarization. STAT1 affects polarization, with a supporting pro-inflammatory phenotype [70], while STAT3 plays a dual role in macrophage polarization, contributing to the regulation of M1/M2 balance. However, its activation mostly promotes M2 polarization [71]. Nuclear factor erythroid 2-related factor 2 has a neuroprotective effect following TBI by suppressing the inflammatory response, partly through regulating microglial function [72]. On the contrary, NADPH oxidase appears to promote the polarization toward M1-like microglia, exacerbating neuroinflammation. NADPH oxidase inhibition was reported to suppress M1-mediated neuroinflammation and shift the M1/M2 balance toward the M2 phenotype after TBI [73].

Evidence from TBI mouse models also highlights the involvement of the nucleotide-binding oligomerization domain-, leucine-rich repeat-, and pyrin domain-containing 3 (NLRP3) inflammasome/gasdermin D axis in post-TBI microglia-mediated neuroinflammation [74]. Activation of this axis promoted the release of neutrophil extracellular traps, further activating microglia, enhancing proinflammatory cytokine release, exacerbating blood–brain barrier (BBB) damage, and worsening neurological deficits. Pharmacological inhibition of NLRP3 and gasdermin D, on the other hand, significantly suppressed neutrophil extracellular trap formation, decreased microglial polarization, and mitigated both the neuroinflammatory response and the resulting neurological deficits. Hypoxia-inducible factor 1 alpha (HIF-1α) has been proposed to contribute to NLRP3 inflammasome-dependent pyroptosis, the release of inflammatory factors, and brain tissue damage after TBI [75]. HIF-1α promoted microglial activation after TBI, as evidenced by the loss of the resting ramified morphology and the adoption of a rounded, activated phenotype. This activation was inhibited following HIF-1α inactivation. Another axis discussed in relation to the NLRP3 inflammasome is the phagocyte NADPH oxidase 2–reactive oxygen species (ROS)–NLRP3 axis [76]. In vitro, NADPH oxidase 2 inhibition dose-dependently reduced ROS signaling, NLRP3 inflammasome-mediated production of proinflammatory cytokines, and pyroptotic cell death [76].

Disease-associated microglia (DAM) are a microglial subtype identified within several microglial clusters, including those related to AD [77]. A TBI mouse study found DAM-like cells in the acute phase, and this transition appeared to depend on the triggering receptor expressed on myeloid cells 2 (TREM2) [78]. TREM2-modulated microglial glycolysis was further identified as a possible partial contributor to this acute-phase DAM transition. In another study in TBI mice, endogenous TREM2 peaked at 3 days after injury and was predominantly localized to microglia and neurons [79]. TREM2 activation through injection of an apolipoprotein (Apo)E-mimetic peptide suppressed microglial activation, neutrophil infiltration, and neuronal apoptosis at the injury site, while attenuating BBB disruption and brain edema. Findings from another study corroborated this result, reporting that TREM2 peaked at 3 days after TBI and was mainly expressed on white matter microglia [80]. TREM2 depletion aggravated post-TBI white matter injury by impairing microglial phagocytosis, increasing oligodendrocyte apoptosis and decreasing oligodendrocyte renewal, whereas its upregulation alleviated post-TBI white matter injury.

#### 4.1.2. Persistent and Disseminated Neuroinflammation Post-TBI

The post-TBI neuroinflammatory responses may persist chronically and thus contribute to further neurodegeneration [81]. In an experimental study on closed-head injury TBI mouse models, activation of microglia and astrocytes was observed acutely, persisting chronically for more than a year post-injury, in addition to elevated infiltration of peripheral immune cells, including lymphocytes [81]. Furthermore, these inflammatory responses were found in regions distant from the primary injury, on both the ipsilateral and contralateral hemispheres, indicating a global inflammatory response. Findings from another study on cortical TBI-rat models showed glial-specific pathology on both the ipsilateral and contralateral hemispheres 7 days post-TBI, indicating widespread inflammatory effects despite focal injury [82]. Sustained microglia activation for up to a year, along with progressive post-TBI neurodegeneration, was shown in another study on TBI-mouse models subjected to controlled cortical impact [83]. In this study, chronically activated microglia were demonstrated to be bushy-like, expressing elevated levels of major histocompatibility complex II and NADPH oxidase, particularly at injury margins. Neuroimaging and histological analyses of a single severe TBI induced by controlled cortical impact in mice documented early gray matter gliosis in the injured hemisphere, interestingly, followed by progressive white matter loss and gliosis spread to the contralateral hemisphere and axonal loss at 12 months [84].

Corroborative findings were reported across post-mortem human studies. Microglia continued to stay chronically activated for many years following a single moderate to severe TBI [85]. Some microglia progressively changed into an amoeboid, phagocytic phenotype that expressed markers matching antigen-presenting cells, up to 18 years. Interestingly, these areas with inflammatory responses were associated with white matter injury and loss of myelin in the corpus callosum, many years after the injury. Diffuse neuroinflammatory responses were also evidenced in another human post-mortem study, marked by activated microglia and astrocytes across both the ipsilateral and contralateral hemispheres [86].

### 4.2. Apolipoprotein E and Polygenic Risk

#### 4.2.1. The Role of ApoE4 in AD Risk Following TBI

The presence of the ApoE4 allele may serve as a predictive marker in the prognosis of TBI patients [87]. An investigation showed that older adults with the ApoE4 allele had a 2-fold increased risk of AD, while those with the ApoE4 allele and a history of TBI had a 10-fold higher risk of developing AD, proposing a synergistic link [88]. However, head injury alone was not associated with increased AD risk. ApoE4 allele carriers tend to have poorer TBI outcomes over a six-month course [89]. Likewise, individuals with the ApoE4 genotype were found to be two times more likely to experience poor six-month outcomes following head injury, compared to those without ApoE4 [90]. A systematic review found that the influence of the APOE ε4 allele on outcome differed by injury severity; in mild TBI, 58.3% of studies concluded the association as non-contributory, 37.5% hazardous, and only 4.2% protective, whereas in severe TBI, 63.6% of studies concluded it was hazardous, 27.3% non-contributory, and 9.1% protective [91]. On the other hand, some previous studies demonstrated contradictory results, indicating an increased risk of AD in individuals without ApoE4 [92] or no significant genotype influence on the patient’s progress following TBI [93,94].

#### 4.2.2. Mechanisms Linking ApoE, TBI, and AD

Several mechanisms have been proposed to explain how ApoE links TBI and AD. ApoE isoforms influence several functions, such as inflammatory responses, and lipid metabolism [95], synaptic plasticity [96], and oxidative stress [97]. ApoE4 impacts the Aβ pathway by impairing Aβ clearance [98], promoting its production [99], and aggregation [100]. Thereby, it appears that ApoE4 changes the physiological Aβ pathway equilibrium toward Aβ accumulation and plaque formation. Higher levels of ApoE4 during the early seeding of Aβ pathology can lead to Aβ aggregation [101]. The ApoE genotype is also observed to affect the clearance of toxic proteins from the brain, such as tau [102].

The synthesis of ApoE is enhanced by both glial cells and neurons after brain injury [103]. ApoE4 carriers are more likely to show Aβ deposition after TBI [104]. In addition, a study in AD-transgenic mice supported the hypothesis that ApoE4 may promote AD-like pathology following TBI, possibly through interactions with Aβ [105].

In addition, ApoE and/or repetitive head trauma (both independently and synergistically) disrupt BBB integrity, proposing a detrimental impact on tau clearance from the brain [102]. In AD transgenic mice, ApoE4 was observed to promote tau accumulation in somatodendritic and intra-axonal compartments and, therefore, may play a role in determining the severity of axonal injury after acute TBI [106].

Another molecular pathway was proposed in an experimental study linking the ApoE4 genotype to neurodegeneration following repetitive blast-induced TBI [107]. In this study, following repetitive blast exposure, ApoE4 mice showed a significant decrease in phosphatidylinositol 4,5-bisphosphate (PIP2), more dominantly in the hippocampus. Conversely, ApoE3 mice showed maintained or elevated PIP2 levels. This contrast is due to Synaptojanin 1 downregulation. This downregulation does not occur in ApoE4 mice, resulting in low PIP2 concentrations [107]. Thus, when Synaptojanin 1 is high (breaking down PIP2), glycogen synthase kinase-3 beta (GSK3β) remains active, and tau is hyperphosphorylated. ApoE4 expression also exacerbates tauopathy by promoting pathological tau misfolding, as well as promoting tau aggregation and NFT formation [108].

The interaction of microglia and inflammatory cytokines with the pathology of AD also depends on the ApoE4 status [109]. Several cytokines, including IL-10, IL-13, IL-4, and IL-1α, were associated with reduced tau pathology only in non-carriers, while in carriers, tau burden increased with microglial density. Other findings propose that ApoE4 carriers tend to show a shift in microglia towards a proinflammatory state [110].

#### 4.2.3. Polygenic AD Risk

Late-onset AD is genetically complex. In addition to ApoE, several other genetic loci underlying AD have been identified [111]. For instance, a specific TREM2 variant was found to confer a significant risk of AD [112]. A TBI mouse study of tauopathy indicates that TREM2 deficiency possibly enhances chronic inflammation and accelerates neurodegeneration [113]. The findings revealed decreased myeloid cell activation surrounding the damaged tissue in TREM2-deficient tauopathy mouse models shortly after the injury (3 days). In the chronic phase (120 days), however, these mice showed macrophage accumulation within the white matter tracts of the corpus callosum, worse axonal injury, tau aggregation, and disrupted neurogenesis. Moreover, TREM2 deficiency compromised microglial phagocytosis of degrading neurons and the restoration of BBB integrity, leading to white matter neurodegeneration and ongoing BBB leakage. In a controlled cortical impact model of TBI, TREM2 activation by an ApoE-mimetic peptide alleviated the induced neuronal damage in mice [79]. However, not all evidence points toward TREM2 upregulation being beneficial after TBI. In a study focusing on the subacute phase after injury, TREM2 upregulation led to excessive microglial phagocytosis and synaptic loss, while its selective knockdown reduced microglia-mediated phagocytosis of synapses and enhanced synaptic plasticity [114]. The role of TREM2 after TBI therefore appears to be phase- and context-dependent.

The clusterin (CLU, also known as ApoJ) gene is reported to be associated with AD risk in a genome-wide association study [115]. With respect to TBI, examination of post-mortem brain tissue from both acute and chronic head-injury cases has demonstrated a significant role for CLU in regulating inflammatory responses during both phases [116]. The pronounced accumulation of CLU within astrocytes, occurring in parallel with progressive neuroinflammation and microglial activation, supported its potential contribution to TBI-associated neurodegeneration. Elsewhere, an experimental study using a rat model of lateral fluid-percussion TBI explored CLU as a candidate biomarker for the diagnosis of acute TBI [117]. CLU protein showed a prolonged, region-specific extracellular upregulation, consistent with CLU protein and mRNA levels in CSF but not with plasma levels, which instead showed an early decrease.

Bridging integrator 1 (BIN1) is another gene showing a significant association with AD in a genome-wide association study [118]. Its mechanism has been linked to tau-related pathology, evidenced by accelerated tau aggregation and propagation [119]. Separate findings support TBI as a risk factor for tauopathies, because TBI has been shown to induce tau hyperphosphorylation and aggregation in tau transgenic mice [120]. However, whether the BIN1–tau relationship extends to TBI-related tau pathology is unclear, as the role of BIN1 in TBI has not been well studied in the existing literature.

### 4.3. Amyloid- and Tau-Related Pathologies After TBI

Aβ is a small peptide fragment from a larger transmembrane protein known as amyloid precursor protein (APP). Aβ accumulation forms dense plaques in the brain, which, together with intracellular tau tangles, are often described in association with AD. Aβ accumulation is by many considered neurotoxic, leading to synaptic dysfunction and eventually, neural death. APP was found to co-accumulate with beta-site amyloid precursor protein cleaving enzyme (BACE) and presenilin-1 (PS1) as well as with Aβ in the damaged axons of post-mortem TBI brain tissue [121]. Progressive aggregation of these peptides into oligomers, fibrils, and amyloid plaques further triggers neurotoxicity and contributes to AD development. Tau is a microtubule-associated protein mainly in neurons, where it stabilizes and assembles microtubules, particularly in axons. Normally, tau supports synaptic integrity and function, but when hyperphosphorylated, it is demonstrated to associate with early neurodegeneration and synaptic loss, independently of seed-competent tau aggregates [122].

Amyloid- and tau-related pathologies appear to be the main molecular links between TBI and later neurodegenerative disorders, including AD. As suggested by a review study, the relationship between TBI and Aβ/tau pathology is multifactorial and dynamic, influenced by pre-existing changes in Aβ and tau biology, secondary immune responses, and age-related alterations in microglial and macrophage function [123].

#### 4.3.1. Human Neuropathology

Histopathological findings from post-mortem studies demonstrated that TBI can be associated with protein-related changes, including Aβ plaques and NFTs, even in younger individuals [124]. Examination of 16 post-mortem severe TBI cases revealed Aβ deposits in 38% of brains, within days of injury [125]. In a larger series of head-injured cases compared to neurologically normal controls, Aβ accumulation was observed in 30% of the head-injury subjects, with its extension and frequency associated with age [124]. Increased βAPP immunoreactivity was found in the neuronal cell bodies (perikarya) near the Aβ deposits, appearing to possibly initiate an AD-type process in the acute phase post-injury. Aβ deposits were also detected in some younger individuals, as young as 10 years old. It seems that while age can increase the chance of Aβ deposition, it is not a necessity for its formation after head injury. Another study examined the temporal cortex of patients with severe TBI undergoing neurosurgery and found that diffuse Aβ deposits formed rapidly, within hours, in approximately one-third of patients [126]. In these plaque-positive cases, enhanced neuronal APP immunostaining was the only significant cellular alteration detected. NFT-like alterations were detected in only two cases. Both cases were of advanced age and did not have Aβ deposits. It appears that tangle formation results from a more chronic process. Unlike AD, dense-cored Aβ plaques and NFTs were rarely observed in TBI cases, suggesting that while there is a shared pathology, each condition has its distinct features.

Notably, Aβ pathology is not limited to the acute post-TBI phase. A study on the brains of long-term survivors of a single TBI showed widespread tau and Aβ pathology, suggesting a chronic link between TBI and the development of neurodegenerative diseases such as AD [127]. This study demonstrated NFT pathology more extensively and at a higher density in single TBI survivors of more than a year than in age-matched controls, commonly in superficial cortical layers. The incidence of Aβ plaques in TBI survivors was similar to that observed in controls. However, the plaques differed in nature, as thioflavin-S staining showed that most TBI cases appeared to have fibrillary or mixed fibrillary/diffuse plaques. Moreover, analysis of the post-mortem TBI brains of late survivors of a single moderate or severe TBI showed widespread hyperphosphorylated tau pathology, which appeared to increase with advanced age [128].

βAPP is proposed as a general marker of axonal injury, identifying damaged axons within white matter tracts [129]. Examination of post-TBI brain tissues found APP to co-accumulate with BACE and PS1, as well as their cleavage product Aβ [121]. Tau protein accumulation, although in only two cases, was also detected within axons and neuronal cell bodies.

Low clearance is another mechanism of Aβ accumulation post-TBI and may reflect broader disruptions in homeostatic regulation following injury. Neprilysin, a zinc-dependent metalloprotease enzyme, plays an important role in the degradation of Aβ [130,131]. A GT repeat polymorphism in the neprilysin gene promoter was associated with Aβ plaque deposition after TBI, with longer GT repeats observed in Aβ-accumulators than in non-accumulators [130]. In a post-mortem TBI brain study of 23 cases surviving up to 3 years, Aβ accumulation was found in damaged axons after TBI in both short- and long-term TBI cases; nonetheless, Aβ plaques were mostly absent in the TBI brain of those who survived longer [131]. Post-TBI neprilysin accumulation was also proposed as a potential mechanism that may explain the regression of amyloid plaques over the course of time.

Tau-related pathology is thought to result from the imbalanced regulation of kinase and phosphatase activity, leading to excessive tau phosphorylation or adaptive cytoskeletal remodeling in response to injury-related stress. Thus, TBI can trigger a cascade of biochemical reactions that interfere with these processes. According to the analysis of human brain tissues of patients undergoing surgery for TBI, significantly higher levels of phosphorylated tau were observed in patients with severe and extremely severe TBIs compared to those with moderate injuries [132]. In addition, the associated dysregulation of GSK3 and protein phosphatase 2A in the hyperphosphorylation process of tau was proposed to be in parallel with the severity of the TBI.

#### 4.3.2. Human Biomarkers

Human post-TBI evidence has reported CSF changes in the proteins involved in AD pathology. A systematic review and meta-analysis of 29 studies underscores that the temporal dynamics of fluid biomarkers possibly reflect distinct post-TBI cognitive impairment pathophysiological processes in a stage-specific manner [133]. Moreover, this review found that among the fluid biomarkers, neurofilament light chain, glial fibrillary acidic protein, and total tau had consistent associations with post-TBI cognitive impairment and brain atrophy across different injury severity and stage.

A study on patients with severe TBI found significantly lowered CSF Aβ42 levels compared to control groups at all time points, with lower concentrations associated with poorer outcomes [134]. In this study, CSF tau levels were elevated early post-TBI, but decreased rapidly, showing normal levels in patient samples collected more than six weeks after trauma. Investigation of the time-related Aβ42 levels in CSF and plasma of patients with severe TBI showed contrasting dynamics between CSF and plasma Aβ42 concentrations early after TBI, as decreased CSF Aβ42 was observed with increased plasma Aβ42 levels, both showing a strong correlation with mortality [135]. Veterans with a history of previous TBI showed higher CSF phosphorylated and total tau concentrations, compared to military controls. Aβ42 CSF levels, on the other hand, did not differ between the groups [136]. Increased CSF levels of total tau, neurofilament light chain, S-100B, and glial fibrillary acidic protein were also reported in Olympic boxers within days, despite normal clinical and neurological examination [137]. It is worth noting that neurofilament light chain and glial fibrillary acidic protein remained increased after the resting period, possibly suggestive of ongoing degeneration. Despite the potential role of tau as a fluid biomarker in reflecting pathologies related to post-TBI cognitive dysfunction, a systematic review and meta-analysis discussed that not enough evidence adequately supports its ability to differentiate the cognitive impairment subtypes [133]. Moreover, evidence proposes that an individual’s genetic susceptibility may influence post-TBI amyloid biomarker changes. In veterans with a history of TBI, higher polygenic risk for AD was associated with lower CSF Aβ42/40, with exploratory analysis suggesting that this association may be stronger with more severe injuries [138].

Collectively, changes in the fluid biomarker levels appear to be a key aspect of the early detection and clinical course of post-TBI cognitive impairment and AD-related pathology. However, when explored systematically across the clinical context, no individual biomarker assessment appeared to be adequate [133]. Combining multiple biomarker panels may improve risk stratification and prediction of cognitive outcomes [133].

#### 4.3.3. Human Imaging Studies

Evidence from neuroimaging studies provides a more complex picture. A Positron Emission Tomography (PET) and Magnetic Resonance Imaging (MRI) study using Pittsburgh Compound B detected increased Aβ pathology in long-term TBI survivors, with a distribution pattern that overlapped but was distinct from that observed in AD, suggesting an alternative mechanism for amyloid plaque formation [139]. Flortaucipir PET imaging also revealed elevated tau pathology in long-term moderate to severe TBI survivors, which was observed to associate with white matter microstructural damage [140]. Additionally, the observed increase in flortaucipir binding was associated with CSF markers of neurodegeneration, including total and phosphorylated tau, and with the presence of traumatic axonal injury. Elsewhere, a PET imaging study of military instructors exposed to repetitive subconcussive blast injury detected amyloid accumulation in an approximately five-month interval after baseline, which appeared to be significant in the inferomedial frontal lobe, precuneus, anterior cingulum and superior parietal lobule [141]. Age-matched control participants, by contrast, demonstrated no amyloid deposition. However, this result was not supported by the findings of a cross-sectional PET study of long-term (at least 10 years) survivors of moderate-to-severe TBI and demographically matched healthy controls, which found no association between a history of a single moderate-to-severe TBI and a higher long-term burden of Aβ or tau pathology [142]. Furthermore, neither the injury interval nor older age at the time of injury was significantly associated with Aβ or tau burden in this study. PET imaging of clinically normal older adults also showed no association between a remote history of mild TBI and increased cortical Aβ burden [143]. A PET study of veterans found that a history of TBI was not associated with globally higher Aβ or tau-PET load [144]. However, the study demonstrated a relatively greater deposition of both in frontal and parietal regions, which are primarily vulnerable to injury but are usually affected at later stages of AD. In veterans with TBI and/or post-traumatic stress disorder, PET with a tau tracer showed widespread neocortical tau distribution, with the highest accumulation in the TBI-only group, overlapping with both typical and atypical AD patterns [145]. A systematic review of amyloid- and tau- PET studies further concluded that the cingulate gyrus and the cuneus/precuneus are the regions showing the most robust evidence for high Aβ accumulation following TBI, while the medial temporal lobe, entorhinal cortex, precuneus, and cortex have the strongest evidence for elevated tau [146]. It is important to note that considerable conflicting findings were demonstrated across studies, thus limiting the definitive interpretation of the results.

#### 4.3.4. Experimental Evidence of Amyloid- and Tau-Related Pathology After TBI

Results from a study of transgenic AD mice subjected to controlled cortical impact TBI [147] closely mirrored the intra-axonal Aβ aggregation pathology detected in human studies [121,126,131]. Axonal injury appeared essential for Aβ accumulation after controlled cortical impact trauma in transgenic-AD mice, as the amount of Aβ accumulation was related to the severity of the injury, and none was observed in areas without axonal injury [147]. Furthermore, an experimental study on triple transgenic AD mice reported rapid increases in soluble and insoluble Aβ forms in the injured cortex, with injured axons identified as a major site of accumulation [148]. However, the total levels appeared to normalize within days. The findings indicated that the acute formation and aggregation of Aβ into toxic forms following TBI may lead to a secondary injury cascade, which in the long term might contribute to raising AD development risk. Findings from another study using transgenic-mouse models of AD demonstrated accelerated hippocampal Aβ accumulation 28 days following TBI, accompanied by impaired spatial learning, with neither difference present at 7 days, supporting the notion that brain injury may act as a risk factor in accelerating AD progression [149]. Aβ plaques and advanced brain inflammation increased by 24 weeks post-injury in experimental AD mouse models, and diffuse injury resulted in more white matter and axonal damage than focal injury [63]. BACE1 protein (β-secretase) is involved in the processing of APP and is a translation product of the BACE1 gene. Golgi-localized, gamma-ear-containing, ADP-ribosylation factor-binding protein (GGA3) is a protein responsible for the regulation of BACE1. Reduced GGA3 is suggested to be associated with AD pathology, since lower levels of GGA3 result in higher levels of BACE1 [150]. Following TBI, caspase-mediated depletion of GGA3 and its homolog GGA1 causes a loss of control of BACE1 degradation [151]. In the subacute phase, GGA1 levels recover, but mice with reduced GGA3 show sustained BACE1 and Aβ elevation, underscoring the critical role of GGA3 in this process [151].

Experimental investigations of TBI have demonstrated that increased levels of tau oligomers appear shortly after the injury [152]. The increased prevalence of AD years after experiencing TBI appears to involve the post-TBI released tau oligomers, which may seed tau pathology in distant regions from the injury site [153]. In tau transgenic mice subjected to TBI, increased tau pathology was detected in a temporally- and spatially-dependent manner [120]. Moreover, tau burden increased in synaptically connected brain areas distant from the injury. Thus, even a single TBI can induce progressive tau pathology that appears to follow neural connections rather than just the proximity to the TBI location. Supporting this, another study has reported post-TBI tau accumulation across anatomically connected brain regions far from the injury site [147]. A significant increase in phosphorylated tau immunoreactivity was detected in transgenic-AD mouse models subjected to TBI by controlled cortical impact from 24 h post-injury for up to a week [147]. Notably, tau pathology was independent of amyloid-related pathologies. Persistent and progressive tau pathology was found chronically in a mouse model with a single severe TBI, in addition to synaptic damage and memory impairment, which was reported to mirror that of long-term tau pathology seen in human TBI cases from the researcher’s parallel investigation [128]. The tau pathology extended to both the ipsilateral and contralateral hemispheres in a self-propagating manner. A correlation between repeated mild TBI and the following increased activated levels of Asparaginyl endopeptidase in the hippocampus and hyperphosphorylation of tau was also found in transgenic-AD mice with mild TBI [154]. There was also a translocation of inhibitor 2 of protein phosphatase 2A from the neuron nucleus to the cytoplasm, where it colocalized with hyperphosphorylated tau.

A review study concluded that experimental and human studies indicate early calcium disturbances following TBI that may persist and contribute to dysfunction of neurons and glial cells [155]. Long-lasting rise in intracellular calcium levels and changed calcium homeostasis were observed in a rat TBI study [156]. In this study, intracellular calcium elevation persisted for up to a week but then returned to baseline levels by 30 days post-TBI; however, changes in calcium homeostasis remained evident even after a month. Elevated cytosolic calcium concentrations were shown to alter neuronal APP and tau metabolism in cultured neurons, resulting in pathological changes resembling those observed in AD [157].

Supporting details from human and experimental studies of amyloid and tau pathology following traumatic brain injury in Section 4.3.1, Section 4.3.2, Section 4.3.3 and Section 4.3.4 are tabulated in Supplementary Table S1.

#### 4.3.5. Proteinopathies Other than Amyloid or Tau

Altered Aβ and tau biology has increasingly been the focus of the studies on post-TBI neurodegeneration, but growing evidence also proposes additional or mixed proteinopathies in this process. Post-mortem examination of human brain tissue after TBI has revealed that several proteins associated with neurodegenerative disease, including APP, BACE, PS1, Aβ, α-synuclein, and tau, accumulate together within damaged axons [121]. Multiple pathological cascades may therefore be triggered following TBI. For instance, mechanistic evidence has demonstrated the dysregulation of TDP-43 after TBI. A study in non-transgenic mice showed that a single TBI led to progressive TDP-43 mislocalization, not only at the primary injury site but also in distant regions, persisting for up to 180 days post-injury [158]. Excessive TDP-43 production was shown experimentally to worsen AD neuropathology and accelerate synaptic and cognitive decline following TBI [159]. In addition, post-TBI neuroinflammation promoted TDP-43 expression, which in turn triggered tau phosphorylation and formation of Aβ by interacting with GSK3β and BACE1, respectively. α-Synuclein is also altered by TBI. Injury was observed to change the expression and localization of synucleins in in vitro and in vivo experimental models [160]. Additionally, in rat models of controlled cortical impact, chronic TBI led to marked α-synuclein upregulation and abnormal accumulation within the substantia nigra, a region also showing microglial infiltration [161]. According to the observations, it seems that post-TBI neurodegeneration is not a pure amyloid or tau pathology, and that other proteinopathies, including but not limited to TDP-43 and α-synuclein, may also occur following TBI. This is important since not all patients with post-TBI cognitive decline or dementia will present with pure AD pathology.

### 4.4. Vascular Impairment

The cerebrovascular dysfunction mediators are demonstrated to have an intricate and dynamic relationship with the regional spread of Aβ and tau pathology, which begins in the early stages of AD [162]. An important mechanism in this regard is extended cerebral hypoperfusion, in association with pathogenic formation of blood vessels and vascular remodeling. TBI also leads to vascular damage in the brain, including BBB dysfunction, intracranial bleeding, and hypoxia. The neurovascular link between TBI and AD appears to particularly involve BBB disruption and extravasation of plasma proteins as an important mechanism.

#### 4.4.1. Blood–Brain Barrier Dysfunction and Neurodegeneration

The BBB is a highly specialized barrier with selective permeability, composed of tightly connected endothelial cells, pericytes, microglia, astrocyte end-feet, and the capillary basement membrane.

Its breakdown is one of the earliest and most notable pathological events post-injury, which allows serum proteins to infiltrate into the brain parenchyma [163]. Even subtle BBB damage may be long-lasting in mild TBI mouse models, leading to astrocyte dysfunction and potentially impaired neuronal health and function [164]. A swine model of concussion showed multifocal BBB disruption independently in gray matter, but in overlap with axonal damage in white matter regions [163]. The infiltrated serum proteins were detected mostly around blood vessels, particularly at brain structural interfaces where the brain tissue changed, including gray-white interfaces, periventricular regions, and the subpial layer within the cortex. Parallel analyses of post-mortem human brain tissues reported much more extensive infiltration of serum proteins, but almost in a similar pattern to that observed in the pre-clinical models [163]. However, unlike the models, ischemic and hemorrhagic lesions, as well as edema, were detected in human tissues. Findings from human post-mortem brains showed serum protein leakage into the brain tissue both acutely and chronically after a moderate or severe TBI, indicating persistent widespread BBB dysfunction [165]. On the other hand, experimental studies showed BBB disruption to be transient in either closed head injury [166], blast injury [167] or fluid percussion models [168], which was also observed to be prolonged by hypoxia and hypotension [166]. Another study showed the post-TBI BBB disruption to be biphasic [169]. The delayed BBB breakdown was proposed to depend on other pathways rather than only on the mechanical injury [169]. Importantly, the TBI-induced BBB disruption appeared to be more pronounced in the frontal cortex and hippocampus of mice [169], the latter being one of the earliest brain regions damaged in AD, and particularly vulnerable to insults [170]. Moreover, inflammatory responses along with BBB opening were evidenced after the TBI, marked by microglia activation [167]. Microglia activation was not solely caused by plasma immune cells or components’ extravasation but was mostly a result of processes that occur within the brain tissue. Additionally, TBI increases oxidative stress [167]. A review article suggested ROS as major contributors to BBB breakdown [171]. Likewise, chronic neuroinflammatory responses may potentially play an important role in BBB integrity [172]. Therefore, it appears that the initial mechanistic force is not the only key mediator of post-TBI BBB disruption. Disrupted BBB following mild TBI is closely associated with neuroinflammation in both acute/subacute and chronic phases. However, the crosstalk between post-TBI neuroinflammatory responses and vascular dysfunction is yet to be investigated in relation to injury severity/frequency and different timeframes.

Another important factor in pathological responses after TBI is the matrix metalloproteinases (MMPs), as evidence suggests their importance in post-TBI BBB opening and hemorrhage [173]. Elevated levels of these zinc-dependent proteases lead to several neurological complications through the impairment of angiogenesis, neovascular dysfunction, neuroinflammation, and neurodegeneration [174]. MMP-2 from neurons/astrocytes and MMP-9 from astrocytes are reported to be significantly released after TBI in vivo [175]. Early MMP-9 increase was also documented in the pericontusional brain, while no significant differences were reported in other MMPs (1, 2, and 10) levels [176]. Another study, however, reported increased MMP-7 levels post-TBI and a correlation between its levels and evidence of BBB dysfunction [177].

#### 4.4.2. Cerebral Amyloid Angiopathy and AD

BBB impairment may be an underlying mechanism of Cerebral Amyloid Angiopathy (CAA) [178], characterized by Aβ deposition in the walls of small to medium-sized cerebral vessels. CAA is associated with AD pathology and is usually seen in elderly patients [179]. A review study pointed out that TBI has been introduced as a precursor to neurodegenerative disorders rather than to vascular pathologies, even in studies exploring post-TBI amyloid-related pathology, and that its contribution to CAA remains unexplored [180]. In experimental juvenile TBI models, altered BBB properties and Aβ immunoreactivity were observed in parallel, suggesting that continued phenotypic BBB changes after early brain injury might contribute to toxic product build-up [181]. Moreover, TBI may also cause early alteration in vascular smooth muscle cells of leptomeningeal arteries, including Aβ formation [182]. A significant association is documented between ApoE4 and the presence of CAA after TBI, with an approximately 8 times higher risk of post-TBI CAA in ApoE4 carriers compared to non-carriers [183]. However, no associations are reported between CAA and injury severity.

#### 4.4.3. Post-TBI Intracranial Bleeding

Intracranial bleeding (IB) is another post-TBI vascular pathology that occurs in approximately 46% of TBI cases [184]. IB is classified into subdural hemorrhage, epidural hemorrhage, subarachnoid hemorrhage, and intraparenchymal hemorrhage. Non-traumatic IB is observed to be associated with an elevated, about two-fold, risk of dementia incidence [185]. A systematic review and meta-analysis reported a doubling of the risk of dementia in individuals with cerebral microbleeds compared with those without them [186]. A large-scale study also compared dementia development in individuals with intracranial lesions following moderate-to-severe TBI with that in those with mild TBI (without any intracranial lesion) and demonstrated an association between the former and an elevated future risk of dementia [187]. According to an abstract from the 73rd Annual Meeting of the American Academy of Neurology, published in a supplement to Neurology, the ten-year cumulative dementia incidence and risk were observed to be higher in patients with traumatic intracerebral hemorrhage compared to those who experienced spontaneous intracerebral hemorrhage [188]. It can be thought that other mechanisms occurring following TBI might contribute to this higher risk.

Extravasated blood erythrocytes undergo lysis and release hemoglobin following hemorrhagic brain injury. Hemoglobin subsequently breaks down and leads to the release and accumulation of labile iron in the brain parenchyma. Ferroptosis is an iron-dependent form of programmed cell death. Evidence indicates that ferroptosis may contribute to the pathological mechanisms leading to cognitive impairment and neurodegeneration after TBI, and that its inhibition might be a potential treatment for TBI. Supporting this, an ex vivo and in vivo study demonstrated the involvement of ferroptosis in neuronal death and functional outcome following TBI [189]. Another study showed activated ferroptosis following TBI in a controlled cortical impact mouse model, in addition to reporting iron accumulation, impaired iron metabolism, increased expression of ferroptosis-related genes, decreased glutathione peroxidase activity, enhanced lipid peroxidation, and mitochondrial shrinkage [190]. In this study, intracerebroventricular administration of ferrostatin-1, a ferroptosis inhibitor, attenuated iron accumulation, neurodegeneration, and injury volume, and improved cognitive and motor performances. Also, administration of ferristatin II, an iron uptake inhibitor, in a TBI mouse model downregulated transferrin receptor 1 in neurons, inhibited TBI-induced iron homeostasis imbalance, and decreased TBI-induced lipid peroxidation [191]. Moreover, ferristatin II showed protective effects against post-TBI neuronal injury and neurodegeneration, possibly in part through ferroptosis inhibition. Using a controlled cortical impact mouse model, a study reported neuroprotective effects of ruxolitinib, a drug approved for myelofibrosis, on the post-TBI temporal changes in the molecules associated with ferroptosis [192]. In this study, both ferrostatin-1 and ruxolitinib were associated with decreased neurodegeneration and iron accumulation, as well as improvements in motor and memory deficits. Post-TBI ferroptosis was also corroborated by findings of another study in a repeated mild TBI mouse model, which reported iron accumulation, glutathione peroxidase inactivation, enhanced lipid peroxidation, and mitochondrial shrinkage [193]. Treatment with mesenchymal stromal cells alleviated these effects and decreased neurodegeneration and pathological protein deposition, as well as attenuating persistent cognitive deficits.

#### 4.4.4. Impaired Perivascular (Glymphatic) Clearance After TBI

Using dynamic contrast-enhanced MRI with intrathecal contrast in a rat controlled cortical impact model, substantially impaired glymphatic drainage was shown, which was also aggravated by additional hypothermia [194]. The early decrease in glymphatic clearance, and its exacerbation by hypothermia, showed a linear association with subsequent phosphorylated tau and Aβ accumulation, as well as with cognitive deficits. Another study in mice showed decreased glymphatic function by 60%, persisting for at least a month [195]. Genetic deletion of aquaporin-4 further worsened glymphatic dysfunction following TBI and enhanced neurofibrillary pathology and neurodegeneration in the injured brain. In an experimental TBI mouse model, angiotensin II type 2 receptor activation improved glymphatic influx and clearance, reduced astrocytic and microglial activation, and promoted Aβ removal while attenuating phosphorylated tau accumulation, contributing to improved motor and cognitive recovery [196].

**Figure 1:**
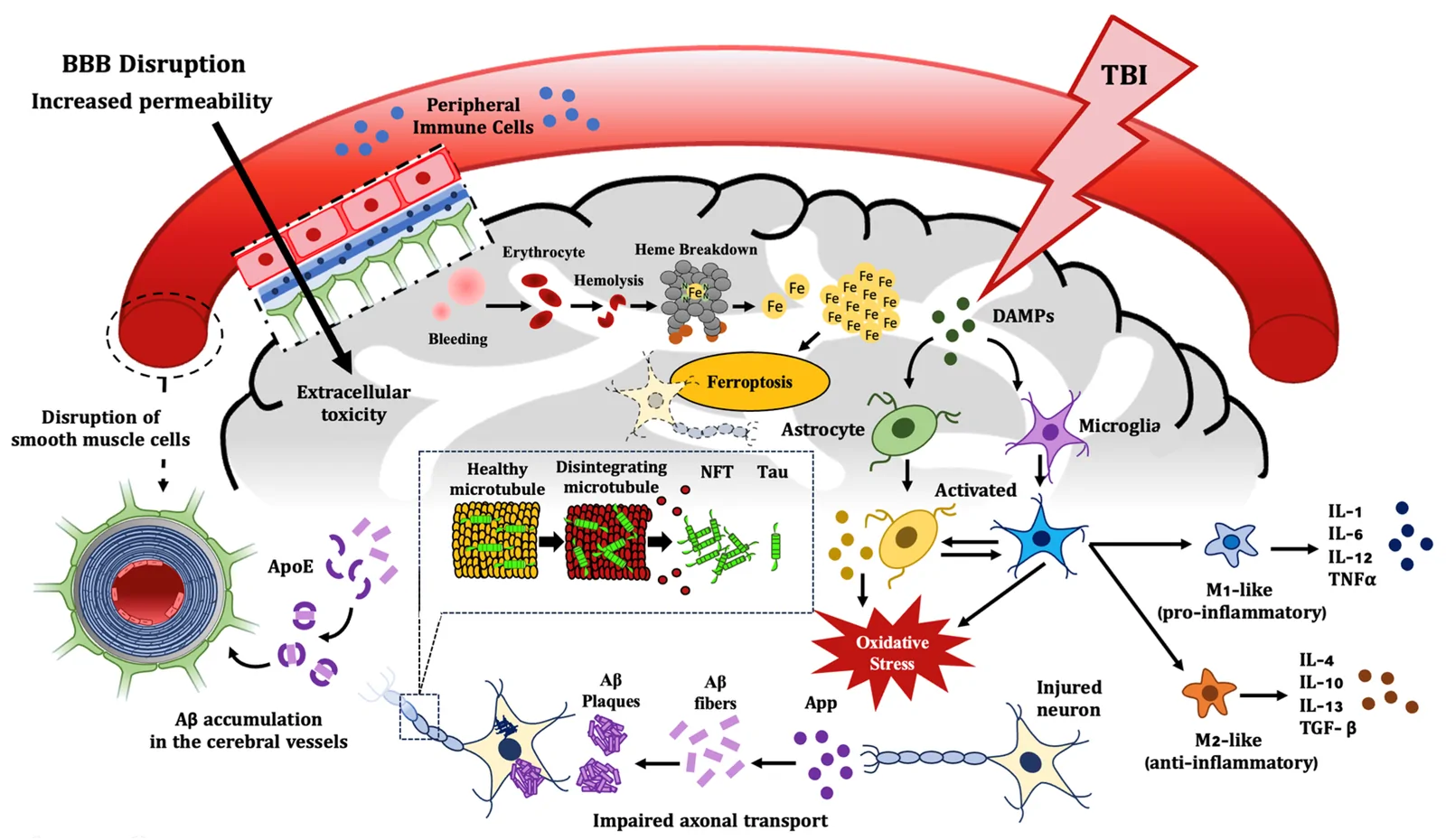
Schematic description of cellular and molecular mechanisms linking TBI and AD. Damage-associated molecular patterns (DAMPs) are released following TBI. Further, these molecules activate resting microglia and astrocytes through pattern recognition receptors. Activated microglia are polarized into at least two phenotypes, M1 and M2, mediated by several signaling pathways. The M1 phenotype exhibits pro-inflammatory responses and expresses cytokines and chemokines such as IL-1, IL-6, IL-12, and TNF-α, while M2 shows anti-inflammatory responses through expression of IL-10, IL-4, IL-13, and TGF-β. Chronic M1 activation results in cytokine release and excessive formation of reactive oxygen species, thus contributing to neurodegeneration. Concurrently, TBI leads to impaired axonal transport of several proteins, which further causes swellings at the site of disrupted transport called axonal bulbs. These proteins’ accumulation is associated with important enzymes that cleave amyloid precursor protein (APP) to Aβ, processes that may reflect injury-related alterations in protein processing and trafficking. Moreover, impaired clearance mechanisms such as neprilysin further promote this aggregation. Injured neurons further degenerate and release the accumulated Aβ into the brain parenchyma, where Aβ plaques may form. Additionally, cytokine signaling leads to dysregulation of kinases and phosphatases, which further results in aggregation of tau in forms of soluble oligomeric and insoluble neurofibrillary tangles. Vascular changes are also among other pathologies related to post-TBI, involving BBB breakdown, hemorrhages, and the infiltration of blood-derived proteins into the brain parenchyma. Importantly, after IB, ferroptosis, an iron-dependent form of programmed cell death, occurs. In addition, dysregulation or persistent microbleeds may sustain oxidative stress and chronic neuroinflammation, which are associated with neurodegeneration.

Figure 1 illustrates a schematic description of cellular and molecular mechanisms linking TBI and AD.

## 5. Conclusions

TBI is associated with an increased risk of dementia and possibly AD, depending on injury severity, frequency, and age at the first trauma. Although this risk may decrease over time, it appears to persist for decades. Shared cellular and molecular pathways, including neuroinflammation, alterations in Aβ and tau homeostasis, and vascular impairment, may represent potential mechanisms linking TBI to AD. Importantly, these pathological changes may reflect complex, context-dependent responses rather than strictly causative processes.

## 6. Future Directions

The utilized TBI severity classification systems differ throughout the studies; therefore, care must be exercised in data interpretation. Moreover, as TBI scaling systems are mostly based on the acute injury characteristics and the individual’s early symptoms, it is difficult to determine the injury outcomes based exclusively on the injury severity. Future research should expand this matter to the frequency of injury and compare outcomes of repetitive mild TBI and a single severe one. Attention should be given to repetitive mild TBI. It may occur frequently in specific populations, such as athletes, but often appears unrecognized, later contributing to cumulative neuropathological changes associated with neurodegeneration. In addition, future research should investigate cellular and molecular pathways underlying the association between AD and TBI, especially the timing of the occurrence of the pathological events, and the potential therapeutic window before these pathologies settle in. This is important because the timing at which therapies targeting each shared pathological mechanism are started might have the potential to influence the development or progression of AD. Longitudinal assessment of plasma biomarkers, particularly different phosphorylated tau species, combined with amyloid/tau PET and other imaging modalities, may offer insight into transient injury-related changes from AD pathology and enable earlier diagnosis and progression tracking of post-TBI AD. Importantly, future research should investigate translational and interventional approaches aimed at modifying the post-TBI neurodegenerative pathways, such as amyloid and tau accumulation, neuroinflammation, and BBB impairment, to name a few. Preclinical and clinical trials targeting these pathways are necessary to determine if initiating treatment at early time points after TBI may reduce the long-term risk of neurodegeneration. Potential factors affecting AD susceptibility, including age, sex, and genetic factors such as APOE genotype, should also be explored more extensively in relation to the association between TBI and later AD.

## Data Availability

No new data were created or analyzed in this study. Data sharing is not applicable to this article.

## Supplementary Materials

The following supporting information can be downloaded at https://www.mdpi.com/article/10.3390/biomedicines14092056/s1, Table S1: Supporting details from human and experimental studies of amyloid and tau pathology following traumatic brain injury in Section 4.3.1, Section 4.3.2, Section 4.3.3 and Section 4.3.4.

## Abbreviations

The following abbreviations are used in this manuscript:

Aβ	Amyloid-beta
Apo	Apolipoprotein
APP	Amyloid precursor protein
BACE	Beta-site amyloid precursor protein cleaving enzyme
BBB	Blood–brain barrier
BIN1	Bridging integrator 1
CAA	Cerebral Amyloid Angiopathy
CSF	Cerebrospinal fluid
CTE	Chronic traumatic encephalopathy
CLU	Clusterin
DAM	Disease-associated microglia
GGA3	Golgi-localized, gamma-ear-containing, ADP-ribosylation factor-binding protein
GSK3 β	Glycogen synthase kinase-3 beta
HIF-1α	Hypoxia-inducible factor 1 alpha
IB	Intracranial bleeding
IL	Interleukin
MMP	Matrix metalloproteinase
MRI	Magnetic Resonance Imaging
NADPH	Nicotinamide Adenine Dinucleotide Phosphate
NFT	Hyperphosphorylated neurofibrillary tangles of tau protein
NLRP3	Nucleotide-binding oligomerization domain-, leucine-rich repeat- and pyrin domain-containing 3
PET	Positron Emission Tomography
PIP2	Phosphatidylinositol 4,5-bisphosphate
PS1	Presenilin-1
ROS	Reactive oxygen species
STAT	Signal transducer and activator of transcription
TBI	Traumatic brain injury
TDP-43	TAR DNA-binding protein 43
TREM2	Triggering receptor expressed on myeloid cells 2
