# Supplementary material for: Traumatic Brain Injury and the Road to Alzheimer’s Disease
Source: Biomedicines. 2026 Sep 13;14(9):2056. doi: 10.3390/biomedicines14092056 (PMC13604123; doi:10.3390/biomedicines14092056)
Supplement: Supplementary file 1 [file biomedicines-14-02056-s001.zip › biomedicines-4446982-supplementary.pdf]

Study (Author, Year)	Study Type; Sample	Key Findings
**Liao et al., 2026** [1]	Systematic review & meta-analysis; 29 studies including 19 cohort, 6 case-control, and 4 cross-sectional studies, with sample sizes ranging from 30 to 472 participants	Neurofilament light chain (NfL), ubiquitin C-terminal hydrolase L1, total tau, and glial fibrillary acidic protein (GFAP) were consistently associated with cognitive impairment and brain atrophy across traumatic brain injury (TBI) severities and stages.<br>Acutely, after the injury (≤7 days), brain-derived neurotrophic factor, neuron-specific enolase, and interleukin-1β correlated with cognitive impairment; in the recovery phase (6 months to 8 years), GFAP and NfL were the significant markers. The study findings highlighted that axonal injury, glial activation, neuroinflammation, neuronal damage, and degeneration drive post-TBI cognitive impairment, with tau pathology and synaptic dysfunction proposed as possible mechanistic links between TBI and AD.
**Dybing et al., 2025** [2]	Systematic review; 26 studies (20 used at least one Aβ tracer, 10 used a tau tracer)	The strongest evidence for raised amyloid-beta (Aβ) was in the cingulate gyrus and cuneus/precuneus, while for tau it was in the medial temporal lobe, entorhinal cortex, precuneus, and the cortical lobes. Results conflicted across most other regions. The authors concluded that there was insufficient evidence to establish whether TBI is associated with Aβ Aβ or tau deposition, though tau findings were the more consistent of the two.
**Neselius et al., 2012** [3]	Prospective cohort study of cerebrospinal fluid (CSF) samples; 30 Olympic boxers (aged 17-34 years) and 25 age-matched non-boxing controls (aged 17-30 years)	CSF concentrations measured within 1–6 days of a bout were higher in boxers than in controls for NfL, GFAP, total tau, and S100B. Phosphorylated tau, Aβ42, and heart-type fatty acid binding protein did not differ. After at least 14 days of rest, only NfL and GFAP remained higher than in controls. NfL correlated with a composite boxing-exposure score.
**Leiva-Salinas et al., 2023** [4]	Prospective cohort neuroimaging study; 9 blast-exposed participants (median age 33 years) and 9 age-matched controls, imaged at two time points (before the exposure, and approximately 5 months after blast exposure)	The control group showed no Aβ deposition, whereas the blast-exposed group showed increased Aβ deposition in four brain regions: the inferomedial frontal lobe, precuneus, anterior cingulum, and superior parietal lobule.
**Hicks et al., 2022** [5]	Cross-sectional study; 87 participants with a history of single moderate-to-severe TBI (mean age 57.53 ± 11.53 years) and 59 controls (mean age 60.34 ± 11.97 years)	PET (Positron emission tomography) imaging showed that a single moderate-to-severe TBI was not associated with increased Aβ or tau pathologies during the chronic phase after the injury. Neither Aβ nor tau burden was significantly associated with time since injury or age at injury.
**Asken et al., 2021** [6]	Cross-sectional and longitudinal amyloid PET neuroimaging study; 134 clinically normal older adults (mean age 74.1 ± 6.8 years), 48 of	A history of remote mild TBI in older adults was not significantly associated with a faster rate of Aβ accumulation compared with those reporting no history of TBI. No interaction was found between mild TBI history and Aβ burden on cognition.

	whom reported remote mild TBI (37±23 years since injury)	
**de Bruin et al., 2025** [7]	Neuroimaging study; 103 male Vietnam War veterans (65 with a history of TBI, 38 without), scanned with florbetapir amyloid PET and flortaucipir tau PET (a mean of 39.6 years after the last injury)	A prior history of TBI was associated with regional differences in Aβ- and tau-PET signal. However, it was not associated with globally increased Aβ- or tau-PET load. In participants with a history of TBI, the relationship between Aβ- and tau-PET was weaker in temporo-parietal regions typical of tau accumulation early in AD, but stronger in regions that typically accumulate tau late in AD.
**Clark et al., 2021** [8]	Cross-sectional secondary analysis of an existing cohort database; CSF samples from 52 cases with TBI and 50 military controls (mean age of 68)	CSF phosphorylated tau and total tau were significantly higher in patients with TBI than in military controls, whereas Aβ did not differ between groups. TBI history moderated the tau-processing speed association.
**Moody et al., 2024** [9]	Retrospective clinical study of CSF samples; total 88 veterans (mean age of 68.3 ± 3.6 years), 49 of whom had a history of TBI	Neither TBI history nor polygenic risk for AD was independently associated with the CSF Aβ42/40, and the two groups did not differ on the ratio itself. However, among veterans with a prior TBI, a higher polygenic risk score predicted a lower Aβ42/40 ratio, whereas no such relationship was seen in those without a TBI.
**Mohamed et al., 2019** [10]	Neuroimaging study; 80 male veterans (10 with moderate-severe TBI, 32 with Post-traumatic stress disorder (PTSD), 17 with both, 21 controls)	Widespread tau accumulation was increased in neocortical regions in all three clinical groups compared with controls, with distributions overlapping both typical and atypical Alzheimer's disease (AD) patterns. The tau deposition was highest in the TBI group. Tau burden correlated with poorer cognitive scores and with cortical Aβ in the PTSD and TBI-plus-PTSD groups, but not in TBI alone.
**Mondello et al., 2014** [11]	Observational study of CSF and plasma samples (pilot cohort); 12 patients with severe TBI (mean age of 49 ± 17.6 years) and 20 controls (average age 26 ± 4 years)	Aβ42 showed opposite patterns after severe TBI, with decreased CSF and increased plasma levels compared with controls. Patients who died after TBI showed lower CSF Aβ42 and higher plasma Aβ42 levels than controls, suggesting that greater injury severity increases the magnitude of these alterations.
**Scott et al., 2016** [12]	Cross-sectional neuroimaging study; 28 participants (9 with a history of single moderate-severe TBI; 11 months to 17 years after injury, 10 with AD, 9 controls)	Long-term TBI survivors showed increased Aβ pathology, with a pattern overlapping with that of AD, but involving the cerebellum unlike AD. The authors suggested that this pathology may be related to the post-TBI axonal injury.
**Gorgoraptis et al., 2019** [13]	Cross-sectional and observational neuroimaging and CSF biomarker study; 21 participants (aged 29-72 years) with a history of single moderate-to-severe TBI, scanned at a median of 32 years after the injury (range 18-51 years)	One-third of the TBI cases showed extensive increases in cerebral flortaucipir binding, one-third showed limited increases, and the remaining third showed no abnormalities. Binding extent correlated with both white matter FA and density, but not with grey matter density. While neither CSF total tau nor phosphorylated tau increased across the TBI cases, CSF total tau correlated with flortaucipir binding in the cerebral cortex, and phosphorylated tau correlated with binding in white matter. The authors concluded that the increase in binding was associated with CSF markers of neurodegeneration, including

		total and phosphorylated tau, and with traumatic axonal injury, which may provide the initial trigger for tau accumulation.
**Franz et al., 2014** [14]	Retrospective analysis of CSF samples; 29 patients with severe TBI, sampled between 1-284 days after injury	CSF Aβ42 was significantly decreased in patients with TBI compared with controls at any time point sampled, with its levels correlated significantly with patient outcome.<br>CSF tau was significantly higher in patients with TBI than in controls; on serial sampling, it rose shortly after injury, peaked at the second week, and then decreased. Tau levels were normal in samples collected more than 6 weeks after trauma.
**Roberts et al., 1994** [15]	Postmortem human brain tissue study; 152 patients (aged 8 weeks to 81 years; survival time 4 hours to 2.5 years) and 44 controls (aged 51-80 years)	Aβ deposits were found in approximately 30% of cases, in one or more cortical areas, and their extent and frequency appeared to increase with advancing age. Increased amyloid precursor protein (APP) immunoreactivity was observed in neuronal perikarya near the Aβ deposits.<br>The authors concluded that APP upregulation occurs as an acute response following brain injury, and that when extensively overexpressed, it promotes Aβ deposition, which may initiate AD-like pathology within days.
**Johnson et al., 2012** [16]	Retrospective postmortem human brain tissue study; 39 patients (aged 19-89 years; survival time 1-47 years) and 47 uninjured age-matched controls	Abundant and widely distributed neurofibrillary tangles (NFTs) were detected in about a third of TBI cases. Among cases aged 60 or younger, NFTs were detected in 34% of TBI cases compared with 9% of controls. Aβ plaque pathology incidence was similar in both groups(28%), while the plaque extent differed significantly. Plaque-positive controls showed predominantly diffuse plaques on thioflavin-S staining, whereas 64% of plaque-positive TBI cases demonstrated positive thioflavin-S staining plaques or a mix of both diffuse and thioflavin-S-positive plaques.<br>The authors concluded that the observed widespread tau and Aβ pathology (years after a single traumatic head injury) may play a role in the development of neuropathologies similar to those of neurodegenerative diseases such as AD.
**Chen et al., 2009** [17]	Postmortem human brain tissue study; 23 cases (aged 16-79 years; survival time up to 3 years) and 3 controls (aged 15-33 years)	Extensive axonal pathology persisted in long-term TBI survivors, remaining detectable years after the initial injury. Damaged axons showed simultaneous build-up of APP together with the enzymes that cleave it (β-site amyloid precursor protein cleaving enzyme (BACE) and presenilin 1 (PS1)) and with the cleavage product, Aβ. Despite the ongoing intra-axonal Aβ accumulation, plaques were absent in long-term survivors. Neprilysin, an Aβ-degrading enzyme, accumulated following injury, providing a possible explanation for why plaques regress or fail to persist over time.
**Uryu et al., 2007** [18]	Observational postmortem human brain tissue study; 18 cases of fatal TBI and 6 control cases)	The majority of brain tissues from individuals with a history of TBI showed axonal pathology, identified by increased immunoreactivity for APP or NF. Co-immunoreactivity between APP and BACE, and also PS1, as well as Aβ, was found in the axonal bulbs. Two-thirds of the cases showed α-synuclein accumulation, predominantly in association with axonal pathology. Tau pathology, however, was found in only two cases in both neurons and adjacent reactive astrocytes. The authors suggested that following TBI, axonal injury may contribute to the pathological accumulation of proteins implicated in neurodegenerative diseases, including AD and synucleinopathies.
**Johnson et al., 2009** [19]	Postmortem human brain tissue study of the hippocampus and	A GT repeat polymorphism in the promoter region of the neprilysin gene was strongly associated with acute Aβ plaque pathology shortly after TBI. Longer combined GT

	cerebellum, with genotyping; 81 cases (aged 0.15-79 years; survival time 4 hours to 25 days)	repeats in the neprilysin promoter (more than 41 repeats) were associated with a higher likelihood of post-TBI Aβ plaque deposition. Specific alleles showed opposite effects: the presence of at least one allele 22 was associated with Aβ plaque deposition, while the presence of one allele 20 decreased the incidence of Aβ plaques.
**Ikonomovic et al., 2004** [20]	Study of surgically excised temporal cortex; 18 patients (aged 18-64 years) [20]	Diffuse extracellular Aβ deposits were detected in one-third of patients, as early as 2 hours after injury. Dense-cored, mature plaques were observed in only one case. NFT-like tau changes were observed rarely (in only 2 patients), both of advanced age. Aβ pathology developed rapidly following injury, along with early degenerative evidence of neurons, whereas NFTs formation was related to a more chronic process.
**Roberts et al., 1991** [21]	Postmortem human brain tissue study; 16 patients (aged 10-63 years; survival time of 6 to 18 days)	Extensive cortical Aβ deposition was demonstrated in 38% of patients, indicating that severe TBI can trigger Aβ deposition acutely, within days.
**Gentleman et al., 1993** [22]	Postmortem human brain tissue study; 11 patients with severe head injury (aged 23-65 years; survival time 12 hours to 10 days)	APP immunostaining was a sensitive, useful marker of axonal injury, detectable even less than 18 hours after injury, unlike silver stains. APP and Aβ did not colocalize at axonal injury sites, consistent with Aβ production occurring at synaptic terminals rather than along the damaged axon.
**Yang et al., 2017** [23]	Study of surgically resected brain tissue from patients operated on after TBI (149 patients: moderate group 46, severe group 49, extremely severe group 47)	Phosphorylated tau, but not total tau, correlated with Glasgow Coma Scale score in patients with TBI. Hyperphosphorylated tau levels were relatively higher in severe and extremely severe TBI cases than in those observed in the moderate TBI group. Activity of glycogen synthase kinase-3 beta and protein phosphatase-2A, the kinase and phosphatase that regulate tau phosphorylation, showed parallel correlations with TBI severity.
**Zanier et al., 2018** [24]	Mixed human–animal translational study. (1) Postmortem human brain tissue; 15 cases surviving at least a year or more following a single moderate or severe closed head trauma (aged 19-89 years) and 15 age-matched controls with no history of TBI or known neurological disease (aged 20-92). (2) Wild-type mouse model of single severe controlled cortical impact or sham; naïve wild-type mice injected with brain homogenate from 12-month post-TBI or sham animals.	(1) A similar proportion of tau pathology was observed in both autopsy groups; however, the TBI group demonstrated greater pathology in both extent and distribution.<br><br>(2) A single severe TBI induced progressive, spreading tau pathology mirroring TBI tau pathology in humans, which progresses from the injury site to both the ipsilateral and contralateral brain hemispheres. The pathology appeared to be transmissible: intracerebral injection into wild-type mice triggered tau aggregation, memory impairment, and synaptic changes.
**Walker et al., 2012** [25]	TBI Mouse model, in vitro neurons, and human postmortem studies	BACE1 rose quickly and substantially in the acute phase after injury, when GGA1 and GGA3 were both depleted; in the subacute phase, its clearance depended on GGA3 alone.

**Watamura et al., 2025** [26]	MAPT knock-in mouse model with frontotemporal dementia-causing mutations (single or double)	Both lines demonstrated diffuse hyperphosphorylated tau in the entorhinal cortex and hippocampus, but neither developed fibrils or seeding activity. Phenotypes were exacerbated in the double-mutant mouse model, corresponding with an increase in 4T:3R tau. Hyperphosphorylated tau accumulation was accompanied by synaptic loss, neurite degeneration, and behavioral abnormalities.
**Edwards et al., 2020** [27]	Tau-transgenic mouse of single controlled cortical impact	Tau transgenic mice subjected to TBI showed accelerated tau pathology across several brain regions relative to shams, and this pathology increased over time. Pathological tau was also present in areas remote from the impact site but synaptically connected to it.
**Tran et al., 2011** [28]	3xTg-AD mouse model of controlled cortical impact	Intra-axonal Aβ accumulation appeared in the pericontusional region of axonal damage, colocalising with the axonal injury markers APP and NfL and correlating with injury severity; none was seen in sham or wild-type animals. Total tau increased with impact depth in the ipsilateral fimbria, with significant increases in the ipsilateral amygdala and contralateral CA1 in moderately TBI mice only. Inhibition of Aβ production by a γ-secretase inhibitor blocked intra-axonal Aβ generation and accumulation almost completely at both 24 hours and 7 days without altering tau pathology, indicating that the two pathologies were accelerated independently after trauma in this model.
**Washington et al., 2014** [29]	3xTg-AD mouse model of controlled cortical impact	Both soluble and insoluble Aβ40 and Aβ42 increased in the ipsilateral cortex at day 1, alongside increased APP processing. Damaged axons were a major site of accumulation. TBI altered the ratio of insoluble to soluble Aβ, so that even after total levels normalised, a greater proportion of Aβ in the injured brain was insoluble. The authors suggested that acute formation of Aβ toxic forms may lead to secondary injury and, chronically, contribute to increased AD risk
**Shishido et al., 2016** [30]	3xTg-AD mouse model of weight-drop	3xTg-AD mice subjected to TBI showed significantly impaired spatial learning and greater Aβ deposition at 28 days after TBI. In contrast, neither spatial learning nor Aβ deposition differed significantly between TBI and sham-treated mice at 7 days.
**Hu et al., 2018** [31]	3xTg-AD mouse model of repetitive mild TBI (5 impacts at 48-hour intervals)	Active asparaginyl endopeptidase was elevated in the brains of mild repetitive TBI mouse models and correlated with tau hyperphosphorylation. Inhibitor 2 of protein phosphatase 2A translocated to the neuronal cytoplasm and colocalised with phosphorylated tau.
**Gerson et al., 2016** [32]	Rat blast or fluid percussion injury models; tau oligomers isolated and injected into hippocampi of human tau mice	Tau oligomers formed after both injury types. Oligomer injection into human tau mice accelerated cognitive impairment onset and increased oligomer levels at both the injected site and in the cerebellum, suggesting seeded spread.
**Hawkins et al., 2013** [33]	Rat parasagittal fluid percussion injury model	Oligomeric and phosphorylated tau were present by 4 and 24 hours post-injury and persisted at 2 weeks in TBI mice but not in sham controls.
**Sun et al., 2008** [34]	Rat fluid percussion injury model	Basal intracellular calcium was increased at 1 and 7 days and had normalised by 30 days, whereas calcium homeostasis impairment remained as long as 30 days post-TBI.
**Pierrot et al., 2006** [35]	Cultured rat cortical and hippocampal neurons expressing human APP	A rise in cytosolic calcium induced changes in neuronal metabolism of APP and tau resembling those found in AD. Increased calcium triggered transient phosphorylation of both tau and APP by cyclin-dependent kinase 5 and glycogen synthase kinase 3, followed

	by calcineurin-mediated dephosphorylation. Intraneuronal Aβ then accumulated and caused neurotoxicity.

Table S1: Supporting details from human and experimental studies of amyloid and tau pathology following traumatic brain injury in subsections 4.3.1-4.3.4.
Abbreviations: AD, Alzheimer's disease; APP, amyloid precursor protein; Aβ, amyloid-beta; BACE1, beta-site APP-cleaving enzyme 1; CSF, cerebrospinal fluid; GFAP, glial fibrillary acidic protein; NfL, neurofilament light chain; NFT, neurofibrillary tangle; PET, positron emission tomography; PS1, presenilin 1; PTSD, post-traumatic stress disorder; S100B, S100 calcium-binding protein B; TBI, traumatic brain injury

Reference

1. Liao, Y.; Zhao, L.; Zhu, Y.; Ye, S.; Huang, J.; Niu, Z.; Zhang, L.; Lei, N.; Guo, P.; Xie, Y. Fluid Biomarkers of Cognitive Impairments Following Traumatic Brain Injury: A Systematic Review and Meta Analysis. *Int J Mol Sci* **2026**, *27*, 4274, doi:10.3390/ijms27104274.
2. Dybing, K.M.; Vetter, C.J.; Dempsey, D.A.; Chaudhuri, S.; Saykin, A.J.; Risacher, S.L. Traumatic Brain Injury and Alzheimer's Disease Biomarkers: A Systematic Review of Findings from Amyloid and Tau Positron Emission Tomography. *J Neurotrauma* **2025**, *42*, 333-348, doi:10.1089/neu.2024.0055.
3. Neselius, S.; Brisby, H.; Theodorsson, A.; Blennow, K.; Zetterberg, H.; Marcusson, J. CSF-biomarkers in Olympic boxing: diagnosis and effects of repetitive head trauma. *PLoS One* **2012**, *7*, e33606, doi:10.1371/journal.pone.0033606.
4. Leiva-Salinas, C.; Singh, A.; Layfield, E.; Flors, L.; Patrie, J.T. Early Brain Amyloid Accumulation at PET in Military Instructors Exposed to Subconcussive Blast Injuries. *Radiology* **2023**, *307*, e221608, doi:10.1148/radiol.221608.
5. Hicks, A.J.; Ponsford, J.L.; Spitz, G.; Dore, V.; Krishnadas, N.; Roberts, C.; Rowe, C.C. β-Amyloid and Tau Imaging in Chronic Traumatic Brain Injury: A Cross-sectional Study. *Neurology* **2022**, *99*, e1131-e1141, doi:10.1212/wnl.0000000000200857.
6. Asken, B.M.; Mantyh, W.G.; La Joie, R.; Strom, A.; Casaletto, K.B.; Staffaroni, A.M.; Apple, A.C.; Lindbergh, C.A.; Iaccarino, L.; You, M.; et al. Association of remote mild traumatic brain injury with cortical amyloid burden in clinically normal older adults. *Brain Imaging Behav* **2021**, *15*, 2417-2425, doi:10.1007/s11682-020-00440-1.
7. de Bruin, H.; Groot, C.; Kamps, S.; Vijverberg, E.G.B.; Steward, A.; Dehsarvi, A.; Pijnenburg, Y.A.L.; Ossenkoppele, R.; Franzmeier, N. Amyloid-β and tau deposition in traumatic brain injury: a study of Vietnam War veterans. *Brain Commun* **2025**, *7*, fcaf009, doi:10.1093/braincomms/fcaf009.
8. Clark, A.L.; Weigand, A.J.; Bangen, K.J.; Thomas, K.R.; Eglit, G.M.L.; Bondi, M.W.; Delano-Wood, L. Higher cerebrospinal fluid tau is associated with history of traumatic brain injury and reduced processing speed in Vietnam-era veterans: A Department of Defense Alzheimer's Disease Neuroimaging Initiative (DOD-ADNI) study. *Alzheimers Dement (Amst)* **2021**, *13*, e12239, doi:10.1002/dad2.12239.
9. Moody, J.N.; Howard, E.; Nolan, K.E.; Prieto, S.; Logue, M.W.; Hayes, J.P. Traumatic Brain Injury and Genetic Risk for Alzheimer's Disease Impact Cerebrospinal Fluid β-Amyloid Levels in Vietnam War Veterans. *Neurotrauma Rep* **2024**, *5*, 760-769, doi:10.1089/neur.2024.0048.

10. Mohamed, A.Z.; Cumming, P.; Götz, J.; Nasrallah, F. Tauopathy in veterans with long-term posttraumatic stress disorder and traumatic brain injury. *Eur J Nucl Med Mol Imaging* **2019**, *46*, 1139-1151, doi:10.1007/s00259-018-4241-7.
11. Mondello, S.; Buki, A.; Barzo, P.; Randall, J.; Provuncher, G.; Hanlon, D.; Wilson, D.; Kobeissy, F.; Jeromin, A. CSF and plasma amyloid-β temporal profiles and relationships with neurological status and mortality after severe traumatic brain injury. *Sci Rep* **2014**, *4*, 6446, doi:10.1038/srep06446.
12. Scott, G.; Ramlackhansingh, A.F.; Edison, P.; Hellyer, P.; Cole, J.; Veronese, M.; Leech, R.; Greenwood, R.J.; Turkheimer, F.E.; Gentleman, S.M.; et al. Amyloid pathology and axonal injury after brain trauma. *Neurology* **2016**, *86*, 821-828, doi:10.1212/wnl.0000000000002413.
13. Gorgoraptis, N.; Li, L.M.; Whittington, A.; Zimmerman, K.A.; Maclean, L.M.; McLeod, C.; Ross, E.; Heslegrave, A.; Zetterberg, H.; Passchier, J.; et al. In vivo detection of cerebral tau pathology in long-term survivors of traumatic brain injury. *Sci Transl Med* **2019**, *11*, eaaw1993, doi:10.1126/scitranslmed.aaw1993.
14. Franz, G.; Beer, R.; Kampfl, A.; Engelhardt, K.; Schmutzhard, E.; Ulmer, H.; Deisenhammer, F. Amyloid beta 1-42 and tau in cerebrospinal fluid after severe traumatic brain injury. *Neurology* **2003**, *60*, 1457-1461, doi:10.1212/01.WNL.0000063313.57292.00.
15. Roberts, G.W.; Gentleman, S.M.; Lynch, A.; Murray, L.; Landon, M.; Graham, D.I. Beta amyloid protein deposition in the brain after severe head injury: implications for the pathogenesis of Alzheimer's disease. *J Neurol Neurosurg Psychiatry* **1994**, *57*, 419-425, doi:10.1136/jnnp.57.4.419.
16. Johnson, V.E.; Stewart, W.; Smith, D.H. Widespread τ and amyloid-β pathology many years after a single traumatic brain injury in humans. *Brain Pathol* **2012**, *22*, 142-149, doi:10.1111/j.1750-3639.2011.00513.x.
17. Chen, X.H.; Johnson, V.E.; Uryu, K.; Trojanowski, J.Q.; Smith, D.H. A lack of amyloid beta plaques despite persistent accumulation of amyloid beta in axons of long-term survivors of traumatic brain injury. *Brain Pathol* **2009**, *19*, 214-223, doi:10.1111/j.1750-3639.2008.00176.x.
18. Uryu, K.; Chen, X.H.; Martinez, D.; Browne, K.D.; Johnson, V.E.; Graham, D.I.; Lee, V.M.; Trojanowski, J.Q.; Smith, D.H. Multiple proteins implicated in neurodegenerative diseases accumulate in axons after brain trauma in humans. *Exp Neurol* **2007**, *208*, 185-192, doi:10.1016/j.expneurol.2007.06.018.
19. Johnson, V.E.; Stewart, W.; Graham, D.I.; Stewart, J.E.; Praestgaard, A.H.; Smith, D.H. A neprilysin polymorphism and amyloid-beta plaques after traumatic brain injury. *J Neurotrauma* **2009**, *26*, 1197-1202, doi:10.1089/neu.2008.0843.
20. Ikonomovic, M.D.; Uryu, K.; Abrahamson, E.E.; Ciallella, J.R.; Trojanowski, J.Q.; Lee, V.M.; Clark, R.S.; Marion, D.W.; Wisniewski, S.R.; DeKosky, S.T. Alzheimer's pathology in human temporal cortex surgically excised after severe brain injury. *Exp Neurol* **2004**, *190*, 192-203, doi:10.1016/j.expneurol.2004.06.011.
21. Roberts, G.W.; Gentleman, S.M.; Lynch, A.; Graham, D.I. beta A4 amyloid protein deposition in brain after head trauma. *Lancet* **1991**, *338*, 1422-1423, doi:10.1016/0140-6736(91)92724-g.
22. Gentleman, S.M.; Nash, M.J.; Sweeting, C.J.; Graham, D.I.; Roberts, G.W. Beta-amyloid precursor protein (beta APP) as a marker for axonal injury after head injury. *Neurosci Lett* **1993**, *160*, 139-144, doi:10.1016/0304-3940(93)90398-5.
23. Yang, W.J.; Chen, W.; Chen, L.; Guo, Y.J.; Zeng, J.S.; Li, G.Y.; Tong, W.S. Involvement of tau phosphorylation in traumatic brain injury patients. *Acta Neurol Scand* **2017**, *135*, 622-627, doi:10.1111/ane.12644.

24. Zanier, E.R.; Bertani, I.; Sammali, E.; Pischiutta, F.; Chiaravalloti, M.A.; Vegliante, G.; Masone, A.; Corbelli, A.; Smith, D.H.; Menon, D.K.; et al. Induction of a transmissible tau pathology by traumatic brain injury. *Brain* **2018**, *141*, 2685-2699, doi:10.1093/brain/awy193.
25. Walker, K.R.; Kang, E.L.; Whalen, M.J.; Shen, Y.; Tesco, G. Depletion of GGA1 and GGA3 mediates postinjury elevation of BACE1. *J Neurosci* **2012**, *32*, 10423-10437, doi:10.1523/jneurosci.5491-11.2012.
26. Watamura, N.; Foiani, M.S.; Bez, S.; Bourdenx, M.; Santambrogio, A.; Frodsham, C.; Camporesi, E.; Brinkmalm, G.; Zetterberg, H.; Patel, S.; et al. In vivo hyperphosphorylation of tau is associated with synaptic loss and behavioral abnormalities in the absence of tau seeds. *Nat Neurosci* **2025**, *28*, 293-307, doi:10.1038/s41593-024-01829-7.
27. Edwards, G., 3rd; Zhao, J.; Dash, P.K.; Soto, C.; Moreno-Gonzalez, I. Traumatic Brain Injury Induces Tau Aggregation and Spreading. *J Neurotrauma* **2020**, *37*, 80-92, doi:10.1089/neu.2018.6348.
28. Tran, H.T.; LaFerla, F.M.; Holtzman, D.M.; Brody, D.L. Controlled cortical impact traumatic brain injury in 3xTg-AD mice causes acute intra-axonal amyloid-β accumulation and independently accelerates the development of tau abnormalities. *J Neurosci* **2011**, *31*, 9513-9525, doi:10.1523/jneurosci.0858-11.2011.
29. Washington, P.M.; Morffy, N.; Parsadanian, M.; Zapple, D.N.; Burns, M.P. Experimental traumatic brain injury induces rapid aggregation and oligomerization of amyloid-beta in an Alzheimer's disease mouse model. *J Neurotrauma* **2014**, *31*, 125-134, doi:10.1089/neu.2013.3017.
30. Shishido, H.; Kishimoto, Y.; Kawai, N.; Toyota, Y.; Ueno, M.; Kubota, T.; Kirino, Y.; Tamiya, T. Traumatic brain injury accelerates amyloid-β deposition and impairs spatial learning in the triple-transgenic mouse model of Alzheimer's disease. *Neurosci Lett* **2016**, *629*, 62-67, doi:10.1016/j.neulet.2016.06.066.
31. Hu, W.; Tung, Y.C.; Zhang, Y.; Liu, F.; Iqbal, K. Involvement of Activation of Asparaginyl Endopeptidase in Tau Hyperphosphorylation in Repetitive Mild Traumatic Brain Injury. *J Alzheimers Dis* **2018**, *64*, 709-722, doi:10.3233/jad-180177.
32. Gerson, J.; Castillo-Carranza, D.L.; Sengupta, U.; Bodani, R.; Prough, D.S.; DeWitt, D.S.; Hawkins, B.E.; Kayed, R. Tau Oligomers Derived from Traumatic Brain Injury Cause Cognitive Impairment and Accelerate Onset of Pathology in Htau Mice. *J Neurotrauma* **2016**, *33*, 2034-2043, doi:10.1089/neu.2015.4262.
33. Hawkins, B.E.; Krishnamurthy, S.; Castillo-Carranza, D.L.; Sengupta, U.; Prough, D.S.; Jackson, G.R.; DeWitt, D.S.; Kayed, R. Rapid accumulation of endogenous tau oligomers in a rat model of traumatic brain injury: possible link between traumatic brain injury and sporadic tauopathies. *J Biol Chem* **2013**, *288*, 17042-17050, doi:10.1074/jbc.M113.472746.
34. Sun, D.A.; Deshpande, L.S.; Sombati, S.; Baranova, A.; Wilson, M.S.; Hamm, R.J.; DeLorenzo, R.J. Traumatic brain injury causes a long-lasting calcium (Ca2+)-plateau of elevated intracellular Ca levels and altered Ca2+ homeostatic mechanisms in hippocampal neurons surviving brain injury. *Eur J Neurosci* **2008**, *27*, 1659-1672, doi:10.1111/j.1460-9568.2008.06156.x.
35. Pierrot, N.; Santos, S.F.; Feyt, C.; Morel, M.; Brion, J.P.; Octave, J.N. Calcium-mediated transient phosphorylation of tau and amyloid precursor protein followed by intraneuronal amyloid-beta accumulation. *J Biol Chem* **2006**, *281*, 39907-39914, doi:10.1074/jbc.M606015200.
